# Podocytes are likely the therapeutic target of IgA nephropathy with isolated hematuria: Evidence from repeat renal biopsy

**DOI:** 10.3389/fphar.2023.1148553

**Published:** 2023-04-07

**Authors:** Mian-Na Luo, Yanqing Yin, Shangmei Li, Junfeng Hao, Cuiwei Yao, Yong-Zhi Xu, Hua-feng Liu, Lawei Yang

**Affiliations:** ^1^ Guangdong Provincial Key Laboratory of Autophagy and Major Chronic Non-communicable Diseases, Affiliated Hospital of Guangdong Medical University, Zhanjiang, China; ^2^ Department of Nephrology, Affiliated Hospital of Guangdong Medical University, Zhanjiang, China; ^3^ Department of the First Clinical Medical College, Guangdong Medical University, Zhanjiang, China

**Keywords:** podocytes, IgA nephropathy, hematuria, repeat biopsy, immunosuppressive treatment

## Abstract

**Background:** The present study aimed to prove the progression of immunoglobulin A nephropathy (IgAN) patients with isolated hematuria based on repeat renal biopsy data for the first time.

**Methods:** 29 IgAN patients with isolated hematuria who received repeat renal biopsies were analyzed retrospectively, while 29 non-isolated hematuria IgAN patients with similar age and background were randomly selected as the control group. Clinical parameters were collected at the time of biopsy. The treatment strategies (conservative treatment with RASS blocker or immunosuppressive treatment) were choosen according to the pathological results at the first renal biopsy. The activity and chronicity indexes of renal lesions were evaluated. Markers of cell inflammation and proliferation were tseted by immunochemistry. The ultrastructure of podocytes was observed by transmission electron microscopy (TEM). Podocyte and oxidative stress marker (NPHS2 and 4-HNE) were detected by immunofluorescence.

**Results:** The IgAN patients with isolated hematuria had better clinical indicators than those with no-isolated hematuria, such as better renal function, higher albumin and lower uric acid. The interval between two biopsies in IgAN patients with isolated hematuria was 630 (interquartile range, 409.5–1,171) days. The hematuria of the patients decreased significantly from 30 (IQR, 4.00–35.00) RBC/ul in the first biopsy to 11 (IQR, 2.50–30.00) RBC/ul in the repeated biopsy (*p* < 0.05). The level of triglyceride decreased significantly (*p* < 0.05). The other clinical indicators were not statistically significant (*p* > 0.05). Deposits of IgA and C3 in the glomerulus were persistent. The activity index decreased, especially cellular crescent formation, while the chronicity index increased. The ultrastructure of podocytes was improved after treatment. The oxidative stress products of podocytes reduced after treatment.

**Conclusion:** Although the clinical indicators of the IgAN patients with isolated hematuria were in the normal range, various acute and chronic pathological changes have occurred, and irreversible chronic changes have been progressing. Cell inflammation and proliferation persisted. Oxidative stress of podocytes was likely to be the therapeutic target. This study provided a strong basis for the progress of IgAN with isolated hematuria through pathological changes before and after treatment. This study will help clinicians recognize the harm of hematuria, change the traditional treatment concept, and help such patients get early treatment.

## Introduction

IgA nephropathy (IgAN) is the first cause of the primary glomerular disease characterized by immunoglobulin A deposition in the mesangial region. IgAN represents more than 40% of the primary glomerular diseases (Li and Liu, 2004) in China. Hematuria is the most typical clinical manifestation of IgAN (Coppo and Fervenza, 2017). More than 70% patients developed gross hematuria or microscopic hematuria after upper respiratory tract infection or intestinal infection (Coppo and Fervenza, 2017). In most patients, microscopic hematuria usually persisted after the disappearance of gross hematuria (Wyatt and Julian, 2013). At present, the pathogenesis of hematuria and its effect on prognosis are still being explored.

It has been reported that in IgAN patients with mild proteinuria (<0.5 g/d), without any intensive treatment, hematuria will naturally decrease, even severe hematuria was not associated with the progression of end-stage renal disease (ESRD) (Tanaka et al., 2015). Therefore, it is traditionally believed that IgAN patients with isolated hematuria have a good prognosis (Brown, 2011), without special treatment, only regular follow-up is required (D'Amico et al., 1981; Barratt and Feehally, 2006). However, recently some scholars have proposed that hematuria is the initial manifestation of IgAN, and proteinuria appears later with the progression of the disease. That is, hematuria is the source and proteinuria is the downstream event. Hematuria may be one of the windows to reflect kidney disease, because it is an important indication of renal inflammatory activity (Vivante et al., 2011). Studies of clinical data from different countries have presented different views, confirming that hematuria and microproteinuria are usually progressive diseases (Lai et al., 2000; Szeto et al., 2001; Li et al., 2002; Gutierrez et al., 2012b). Other scholars found that up to 25% patients with gross hematuria associated AKI could not recover from baseline renal function (1.2 ± 0.3 mg/dL) due to tubular obstruction of red blood cell (Moreno et al., 2012). In fact, many IgAN patients with persistent hematuria are difficult to relieve on their own, thus the patients are very anxious and worried. Clinicians are also very confused. What role does hematuria play in the progression of IgAN? Should we intervene?

In recent years, more and more scholars called on clinicians to pay attention to the influence of hematuria on IgAN. Both prospective clinical cohort studies (Yu et al., 2020) and meta-analysis (He et al., 2021) proved that hematuria was one of the risk factors for progression of IgAN, and both the degree and duration of hematuria affected the prognosis of the disease (Yu et al., 2020). Compared with patients with mild or negative hematuria, the others with persistent hematuria will more easily develop into ESRD over time. Hematuria disappeared in nearly half of the patients during follow-up, and the rate of renal function loss decreased significantly per year (Sevillano et al., 2017). Therefore, hematuria remission can delay the progression of IgAN. So how to relieve hematuria? Some scholars have made a preliminary discussion on the treatment of IgAN patients with persistent hematuria, and believed that RASS blocker can not only reduce proteinuria of IgAN, but also improved the remission rate of hematuria (Chen et al., 2020). Unfortunately, these studies have not been able to dynamically compare clinical and pathological changes in these patients. Although hematuria is an essential symptom of IgAN, few studies have systematically analyzed the effect of persistence and severity of hematuria on the risk of ESRD, and certainly IgAN predictive model indicators do not include hematuria (Barbour et al., 2019).

In our study, we were the first to elucidate the impact of hematuria on the prognosis of IgAN base on repeated renal biopsy. The clinical and pathological data at two renal biopsy punctures was analyzed, and the treatment plan was preliminarily discussed.

## Methods

### Data collection

Patients with biopsy-proven primary IgAN with isolated hematuria were recruited between January 2007 and December 2021 from the medical records at the Affiliated Hospital of Guangdong Medical University (Zhanjiang, China). In brief, the inclusion criteria were as follows: 1) the patients received two renal biopsies, 2) the pathological diagnosis was IgAN, 3) isolated hematuria was the clinical manifestation at the first renal biopsy. Isolated hematuria was defined as hematuria>5 RBC/HP using the manual method and proteinuria with protein excretion ≤0.5 g/d, and 4) after discharge, the patients were regularly followed up to the second renal biopsy. Patients were excluded if they had secondary IgAN (e.g., secondary to purpura nephritis, lupus nephritis, hepatitis B associated glomerulonephritis), systemic diseases, or crescentic IgAN (>50% crescentic glomeruli). Non-isolated hematuria IgAN patients with similar age and background were randomly selected as the control group. In this study, 29 IgAN patients with isolated hematuria and 29 patients with non-isolated hematuria were enrolled. All patients were notified and agreed for their clinical data to be used in this study. The study was conducted in accordance with the Declaration of Helsinki, and approved by the institutional review board of the Affiliated Hospital of Guangdong Medical University.

### Therapeutic intervention

Prior to the first renal biopsy none of the patients received any immunosuppressive agents. As shown in Table 2, most IgAN patients with isolated hematuria after the first renal biopsy were treated with immunosuppressive agents. Similar to the literature (Yu et al., 2020), the immunosuppressive regimen was individualized according to the patient’s histological lesions (whether there are active pathological changes, such as crescent, inflammatory cell infiltration, etc.) and the patient’s informed consent (informing the drug of possible side effects and drug prices). Based on the above consideration, a plan was formulated for each patient. At the same time, the patients were followed up regularly in the outpatient department, and the medication would be adjusted according to the problems in the follow-up process. 25 patients with activity lesion such as cellular crescents, mesangial proliferation and interstitial inflammatory cell infiltration treated with prednisone plus cyclophosphamide (P + CTX), tripterygium wilfordii Hook F (TwHF), azathioprineor (AZA) or mycophenolate mofetil (MMF). Specific dosage and usage of the drugs referred to previous literature (Luo et al., 2014).

### Follow-up

Twenty-nine patients underwent follow-ups regularly at the outpatient clinic until the second biopsy. Clinical symptoms, drug consumption and possible treatment complications of the patients were evaluated during follow-up. Blood pressure was monitored. Laboratory parameters, including serum levels of creatinine, uric acid and albumin, as well as results of routine blood tests were recorded at two renal biopsies. Examination of hematuria in urine was performed using a fully automated urine particle analyzer. The estimated glomerular filtration rate (eGFR) was estimated using the Chronic Kidney Disease Epidemiology Collaboration (CKD-EPI) formula (Levey et al., 2009).

### Renal biopsies and pathological diagnosis

All patients underwent renal biopsies twice under ultrasound guidance. Periodic acid-Schiff metheramine (PASM) and immunofluorescent staining of IgA and complement C3 were performed. The deposition of IgA and complement C3 was semi-quantified as previously described (Luo et al., 2014). According to the 2017 Oxford pathological classification (Trimarchi et al., 2017), the pathological characteristics of the patients were analyzed as follows: mesangial cell proliferation (M0/1), endothelial cell proliferation (E0/1), segmental sclerosis or adhesion (S0/1), renal tubular atrophy or renal interstitial fibrosis (T0/1/2) and crescent body (C0/1/2). The activity and chronicity index were evaluated by the Andreoli scoring method (Luo et al., 2014), and the scores were assessed by two renal pathologists who were blinded to the clinical information.

### Immunochemistry

Kidney biopsies were selected to mark the inflammatory cells and proliferative cells. The following markers were CD3, CD4, CD8, CD20, CD68, CD138 and Ki-67 (all from Dako). The proportion of the positive area was analyzed automatically by ImageJ software as percent of the area of interest (AOD) (Greite et al., 2018).

### Transmission electron microscopy (TEM)

Kidney tissue specimens were fixed, embedded and stained as described previously (Liu et al., 2014). Ultrathin sections were cut using a Philips CM100 electron microscope.

### Immunofluorescence study

Immunostaining analysis for tissues was conducted as described previously (Liu et al., 2014). Rabbit anti-NPHS2 (Abcam ab50339) and mouse anti-4-HNE (Abcam ab48506) antibodies were obtained. Alexa Fluor™ 488 donkey anti-rabbit IgG (Invitrogen, A21206) and Alexa Fluor™ 594 donkey anti-mouse IgG (Invitrogen, A21203) were used for tissue immunostaining assays. Images were taken under TCS SP5 II confocal microscope (Leica Microsystems). Relative fluorescence intensity was calculated by ImageJ software.

### Statistical analysis

Statistical tests were performed with SPSS 26.0 software. Normally distributed variables were presented as mean ± standard deviation (S.D.), and compared using the analysis of variance (ANOVA) test or paired-samples *t*-test. Non-normally distributed data were expressed as median and interquartile range (IQR) and compared using Wilcoxon signed rank test. All categorical data were summarized as frequency or percentage and tested using χ2 test. *p* < 0.05 was considered to indicate a statistically significant difference.

## Results

### General data

Twenty-nine IgAN patients (8 males and 21 females) with isolated hematuria underwent repeat renal biopsies were selected. As the control group, twenty-nine non-isolated hematuria IgAN patients with the same age and background were randomly enrolled. The clinical indicators of IgAN patients with isolated hematuria were better than those patients with non-isolated hematuria (Table 1). For example, IgAN patients with isolated hematuria have lower serum creatinine [(69.0 (IQR, 51.4–84.2) µmol/L vs. 93.0 (IQR, 73.5–126.8) µmol/L] and higher glomerular filtration rate [(106.3 ± 27.3) mL/min/1.73 m^2^ vs. (77.9 ± 31.1) mL/min/1.73 m^2^] than patients with non-isolated hematuria, both of which have statistically significant. The serum uric acid, triglyceride and 24-h urine protein were higher in patients with non-isolated hematuria IgAN (*p* < 0.05), while albumin was lower [(40.8 ± 5.8) g/L vs. (32.9 ± 8.9) g/L, *p* < 0.01] (Table 1).

**TABLE 1:** Comparison of baseline data of patients with isolated hematuria and non-isolated hematuria.

Index	Isolated hematuria (*n* = 29)	Non-isolated hematuria (*n* = 29)	*p*-Value
Age at first renal biopsy (Years), mean (S.D.)	31.0 (10.0)	35.0 (11.7)	>0.05
Gender (Male/Female)	9/20	13/16	>0.05
Systolic pressure (mmHg), mean (S.D.)	125 (15)	126 (17)	>0.05
Diastolic pressure (mmHg), mean (S.D.)	79 (10)	78 (12)	>0.05
Hemoglobin (g/L), mean (S.D.)	123.6 (17.4)	127.5 (18.7)	>0.05
Serum creatinine (µmol/L), median (IQR)	69.0 (51.4–84.2)	93.0 (73.5–126.8)	<0.05^a^
GFR (ml/min/1.73 m^2^), mean (S.D.)	106.3 (27.3)	77.9 (31.1)	<0.01^b^
Uric acid (µmol/L), mean (S.D.)	317.8 (77.8)	430.9 (139.9)	<0.05^a^
Albumin (g/L), mean (S.D.)	40.8 (5.8)	32.9 (8.9)	<0.01^b^
Triglyceride (mmol/L), median (IQR)	1.1 (0.8–1.3)	1.7 (1.1–2.8)	<0.05^a^
Urine protein (g/d), median (IQR)	0.3 (0.2–0.4)	2.6 (2.1–4.2)	<0.05^a^
Hematuria (RBC/ul), median (IQR)	30 (4–35)	20 (10–30)	>0.05

Note: GFR, glomerular filtration rate. Continuous variables were expressed as mean ± S.D., if normal distribution, as median (interquartile range) if non-normal distribution. Categorical variables were expressed as counts. Comparisons were based on Wilcoxon rank-sum test, *t*-test, or chi-square test.

^a^ represented that P was less than 0.05.

^b^ represented that P was less than 0.01.

Isolated hematuria IgAN patients with a mean age of 31 years (range, 18–59 years) underwent their first renal biopsies. The average follow-up days and the durations of the treatments was an interval of 630 (IQR, 409.5–1,171) days between two biopsies. As shown in Table 2, all except four patients received immunosuppressive treatment. The regimen was well tolerated and no severe adverse events were observed during follow-ups.

**TABLE 2:** The treatment of IgA nephropathy patients with isolated hematuria who underwent repeat biopsies (*n* = 29).

Patient	Treatment	Patient	Treatment
1	RASB + PDN + TwHF + AZA	16	RASB
2	RASB + PDN + AZA	17	RASB + PDN + MMF
3	RASB	18	PDN + TwHF + AZA
4	RASB + PDN + TwHF	19	PDF + MMF
5	RASB	20	RASB
6	RASB + PDN + TwHF + AZA	21	PDF + MMF
7	PDN	22	PDF + MMF
8	PDN + TwHF + AZA	23	PDF + CTX
9	RASB + PDN + TwHF + AZA	24	PDF + MMF
10	PDF + MMF	25	PDN + AZA
11	RASB + PDN + AZA	26	RASB + PDN + MMF
12	RASB + PDN + MMF	27	PDF + CTX
13	RASB + PDN + AZA	28	PDF + MMF
14	TwHF	29	RASB + PDN + CTX
15	RASB + PDN + TwHF		

Note: RASB, renin-angiotensin system blockade; PDN, prednisone; TwHF, Tripterygium wilfordii Hook F; AZA, azathioprine; CTX, cyclophosphamideand MMF, mycophenolate mofetil.

### Clinical data of IgAN with isolated hematuria

**FIGURE 1:**
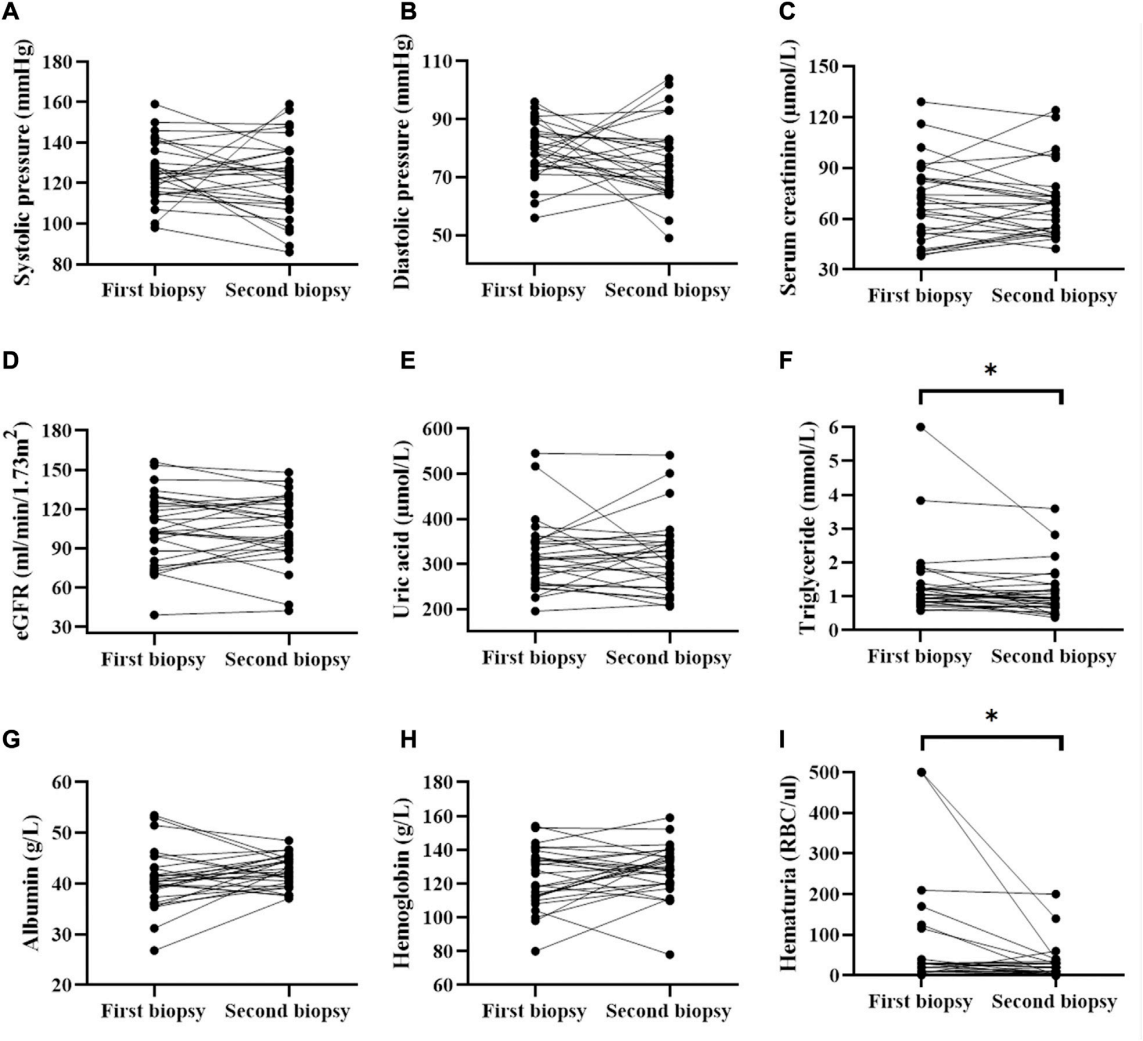
Comparison of clinical parameters before and after treatment (data collected from two renal biopsies). * represented that P was less than 0.05.

Blood pressure, serum uric acid was not significantly different before and after treatment (Figure 1E). Correspondingly, the serum albumin level was (40.8 ± 5.8) g/L and (42.4 ± 3.1) g/L (Figure 1G) before and after treatment. The hemoglobin level was from (123.6 ± 17.4) g/L to (128.3 ± 15.2) g/L. The serum creatinine levels was from 69.00 (IQR, 51.4–84.2) µmol/L to 68.90 (IQR, 52.0–74.2) µmol/L (Figure 1C), while the GFR was from (106.3 ± 27.3) ml/min/1.73 m^2^ to (105.9 ± 25.6) ml/min/1.73 m^2^ (Figure 1D) after treatment. However, there were no significant differences in these clinical indicators between the two biopsies. Additionally, the level of triglyceride decreased significantly from 1.1 (IQR, 0.8–1.3) mmol/L to 0.94 (IQR, 0.7–1.3) mmol/L (*p* < 0.05). Notably, proteinuria occurred in four patients during follow-up (data not shown). The hematuria of the patients decreased significantly from 30 (IQR, 4.00–35.00) RBC/ul before treatment to 11 (IQR, 2.50–30.00) RBC/ul (*p* < 0.05, Figure 1I) after treatment.

### Pathological data

**FIGURE 2:**
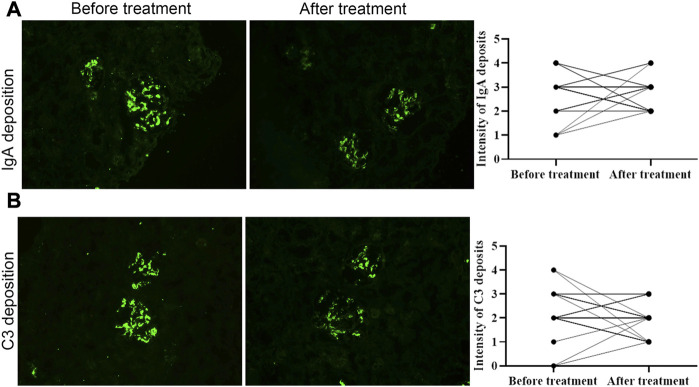
The intensity of immunoglobulin A and C3 deposition before and after treatment. Both of them was not significantly different between two biopsies. Scale bar: 50 μm.

**FIGURE 3:**
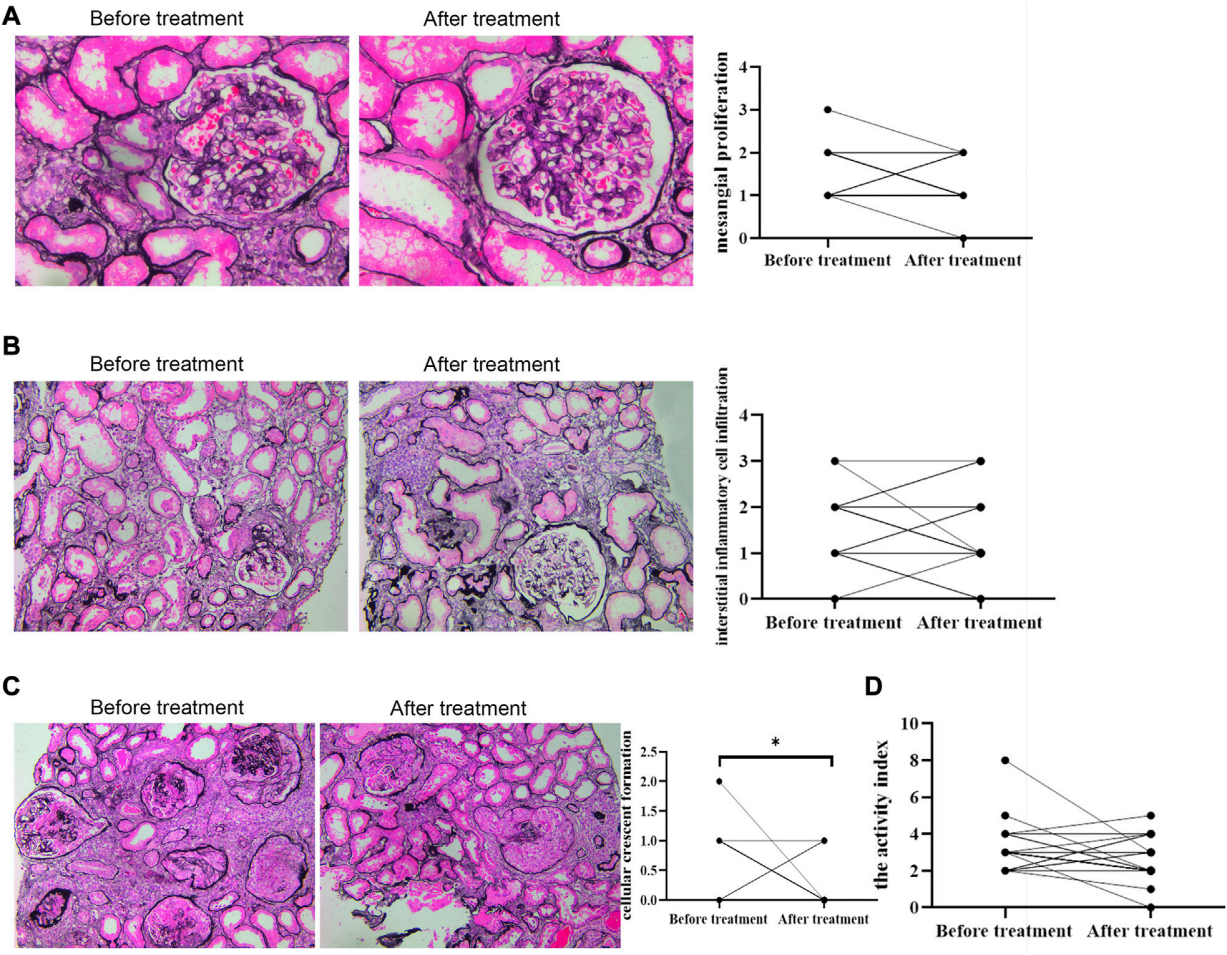
Acute renal pathological changes before and after treatment by PAS staining. Scale bar: 50 μm. Activity indexes of renal lesions were evaluated by Andreoli scoring method. * represented that P was less than 0.05.

**FIGURE 4:**
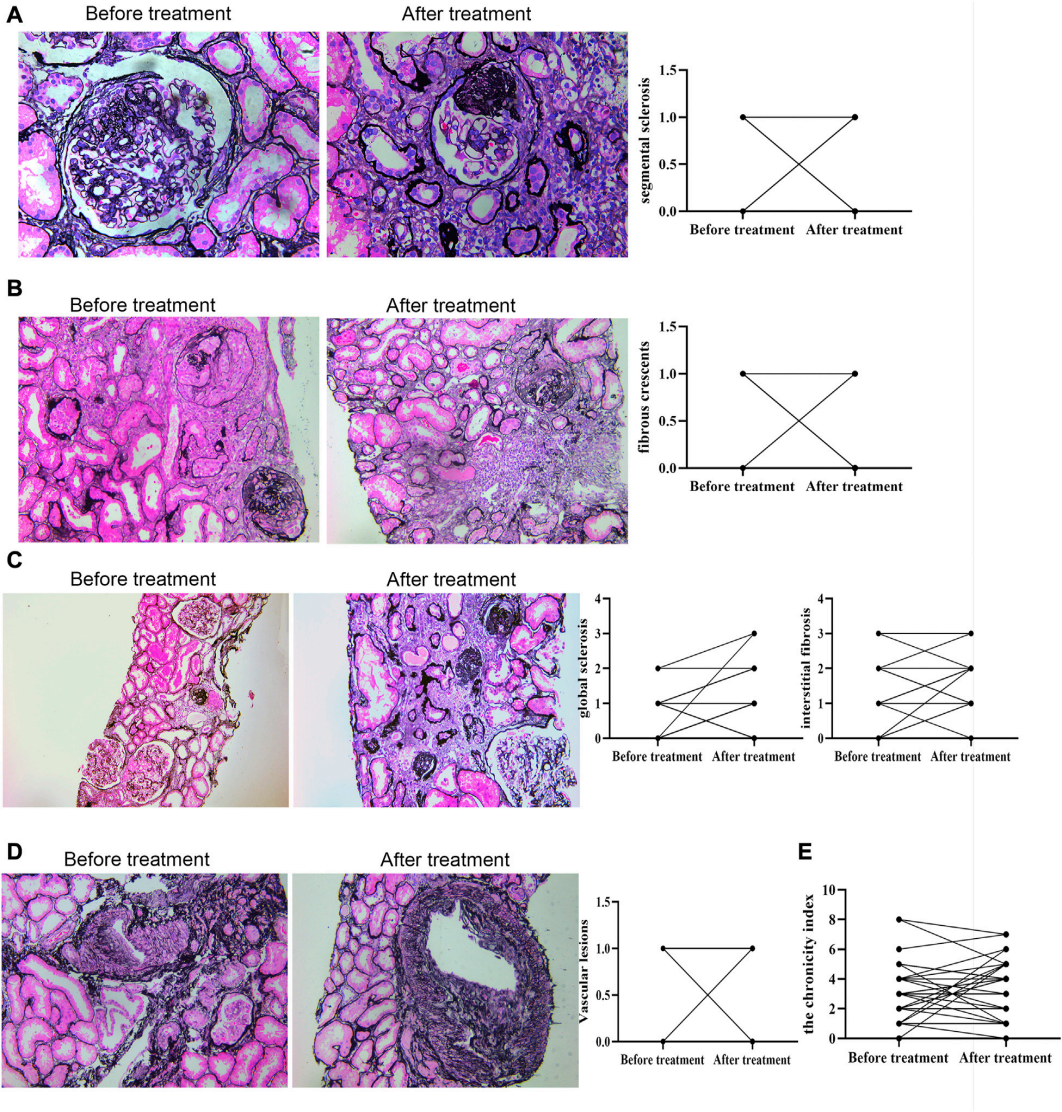
Chronic renal pathological changes before and after treatment by PAS staining. C: scale bar: 100 μm; other figures: scale bar: 50 μm. Chronic indexes of renal lesions were evaluated by Andreoli scoring method.

Deposits of IgA and C3 in the glomerulus were persistent, and C3 deposits were reduced after treatment (Figure 2). The intensity of other immunoglobulin deposition was not significantly different between the two biopsies (data not shown). The distributions of M1, E1, S1, T1-T2, and C1-C2 were 58.62%, 31.03%, 75.86%, 68.97%, and 34.48% before treatment, respectively. After treatment they were 55.17%, 27.59%, 75.86%, 55.17%, and 24.14%, respectively (Table 3). The acute lesions, such as diffuse mesangial (Figure 3A), cellular crescent formation (Figure 3C) or interstitial mononuclear cell infiltrates (Figure 3B) were found in the majority of patients before treatment. The activity index decreased after treatment, especially cellular crescent formation ((Figures 3C, D, *p* < 0.05). In addition, chronic pathological damage, including segmental glomerular sclerosis (Figure 4A), global sclerosis and interstitial fibrosis (Figure 4C), increased after treatment (Figure 4E). Importantly, the fibrous crescents decreased after treatment (Figure 4B). Vascular cavity was larger after treatment (Figure 4D). Because some patients have the same acute and chronic scores, some points in Figures 3, 4 overlap. In fact, one point represented several patients with the same score.

**TABLE 3:** Oxford classification of IgA nephropathy patients with isolated hematuria who underwent repeat biopsies (*n* = 29).

Oxford classification at biopsy	Before treatment (first biopsy)	After treatment (second biopsy)	*P*
M1, n (%)	17 (58.62)	16 (55.17)	>0.05
E1, n (%)	9 (31.03)	8 (27.59)	>0.05
S1, n (%)	22 (75.86)	22 (75.86)	>0.05
T1-2, n (%)	20 (68.97)	16 (55.17)	>0.05
C1-2, n (%)	10 (34.48)	7 (24.14)	>0.05

Note: M, mesangialhypercellularity; E, endocapillary hypercellularity; S, segmental glomerulosclerosis; T, tubular atrophy/interstitial fibrosis; C, crescent.

### Immunohistochemical results of inflammatory cells

**FIGURE 5:**
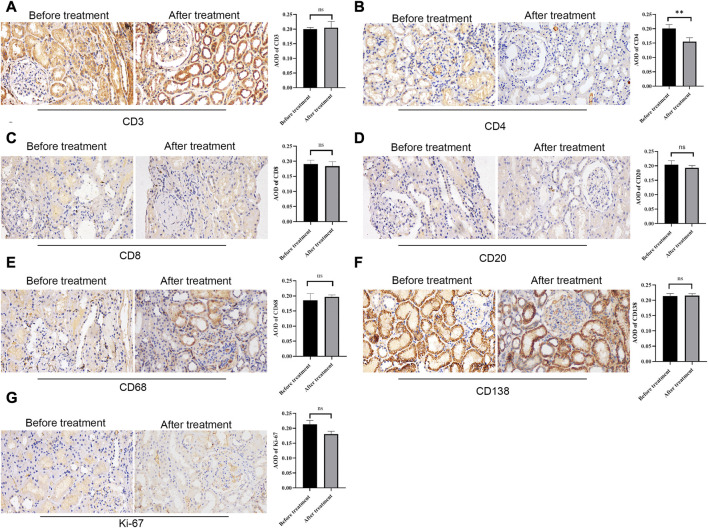
Immunohistochemical staining of CD3, CD4, CD8, CD20, CD68, CD138, and Ki-67 before and after treatment. Representative glomerulus photomicrograph in patients with repeat renal biopsy. Scale bar: 50 μm. ** P < 0.01. ns, no significance.

To observe the expression of inflammatory cells in IgAN patients with isolated hematuria, CD4, CD8, CD20, CD68, CD138 and Ki-67 were performed. The result showed that there were many CD3^+^ (Figure 5A), CD20^+^ (Figure 5D), CD138+ (Figure 5F) and CD68^+^ (Figure 5E) cells infiltrating the tubulointerstitium, however, there was not statistically significant difference between before and after treatment. Interestingly, there was more CD4^+^ cell infiltration before treatment than after treatment (*p* < 0.05, Figure 5B).

### TEM results

**FIGURE 6:**
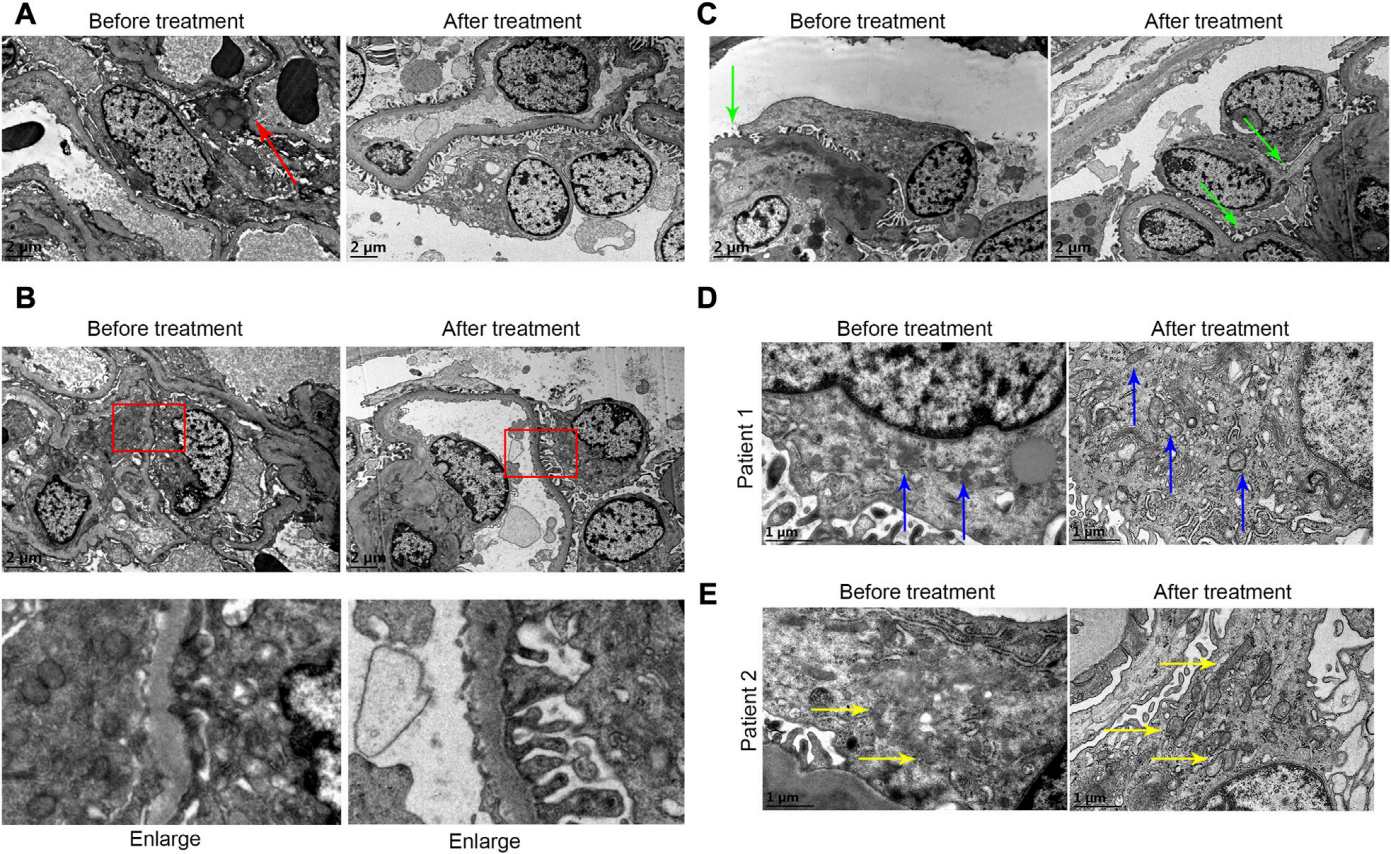
**(A)** In the first renal puncture biopsy specimen, podocytes were flat and apoptotic (as shown by the red arrow) before treatment. The foot cell morphology after treatment was improved. **(B)** It showed foot process fusion of podocytes before treatment. The shape of foot process of foot cells recovered after treatment. **(C)** The podocyte fissure membrane before treatment was more widened before treatment (green arrow). **(D and E)** From patients 1 and 2, respectively. **(D and E)** Before treatment it showed that the mitochondria in podocytes were indistinct, cristae were broken, and local vacuolization. The shape of mitochondria after treatment was improved (blue arrow and yellow arrow).

As shown in Figure 6A, in the first renal puncture biopsy specimen, podocytes were flat and apoptotic (as shown by the red arrow). The podocyte morphology improved after treatment. The first renal biopsy specimen showed foot process fusion of podocytes and the shape of foot process of foot cells recovered after treatment (Figure 6B). The podocyte fissure membrane of specimen was narrowed in the second renal puncture biopsy compared with the first biopsy (Figure 6C, green arrow). The first renal biopsy specimen showed that the mitochondria in podocytes were indistinct, cristae were broken and local vacuolization. The shape of mitochondria after treatment was improved (Figure 6D, blue arrow; Figure 6E, yellow arrow).

### Immunofluorescence results

**FIGURE 7:**
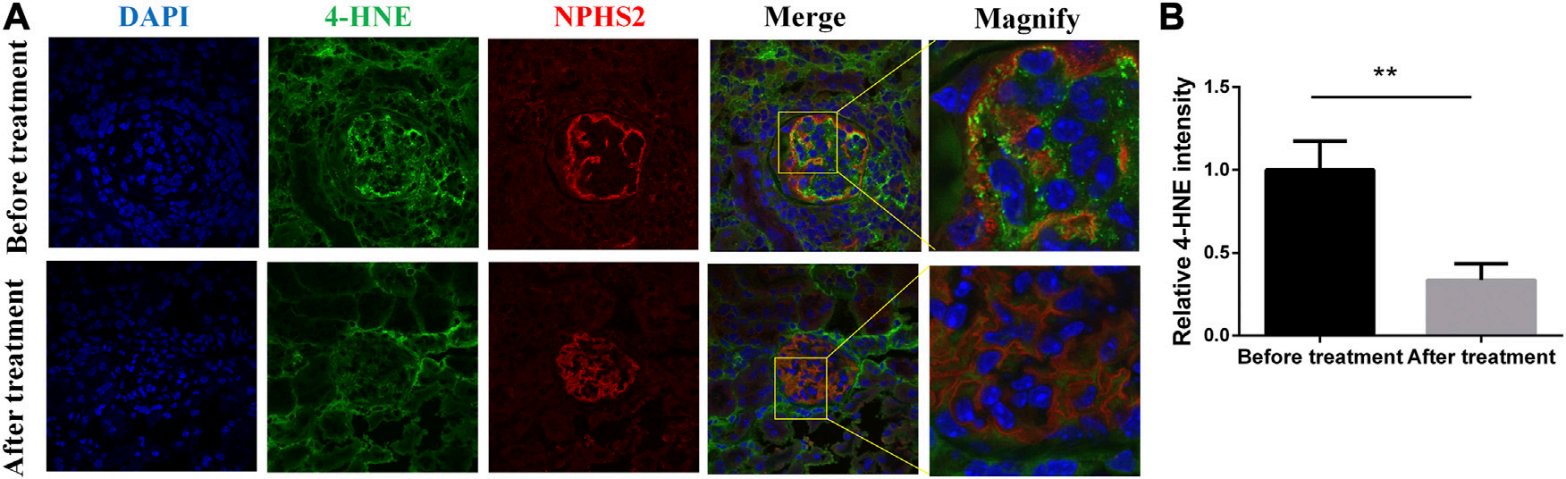
Immunofluorescent staining of 4-HNE and NPHS2 before and after treatment.

As shown in Figure 7, podocyte marker protein (NPSH2, red fluorescence light) increased after treatment, while oxidative stress biomarker (4-HNE, green fluorescence light) decreased after treatment.

## Discussion

Although hematuria is common and even persistent in patients with IgAN, neither the Kidney Disease Improving Global Outcomes (KDIGO) guideline nor previous studies clarified that hematuria is a major risk factor for the progression of IgAN, requiring no special treatment (Barratt and Feehally, 2006). Therefore, many patients with isolated hematuria are not actively undergoing renal biopsy (McGregor et al., 1998; Barratt and Feehally, 2006), resulting in an underestimation of the incidence of IgAN with hemuria (Vivante et al., 2013). In our study, as shown in Table 1, the clinical indicators of IgAN patients with isolated hematuria seemed to be relatively better, such as higher GFR and albumin, lower serum uric acid and triglyceride, stable blood pressure. However, in fact, the pathologic presentation was not as good as expected. Seen from Figures 3–5, the Oxford classification and Andreoli scores suggested acute lesions and renal interstitial inflammatory cell infiltration could be found at the first renal biopsy. Our preliminary results (Luo et al., 2014) also indicated that the clinical and pathological manifestations of patients with IgAN may be inconsistent, so it was crucial to confirm the pathological changes through renal biopsy. The IgAN patients with isolated hematuria can not be ignored, and must be paid sufficient attention.

Up to date, we were the first to compare the pathologic changes of IgAN patients with isolated hematuria through repeated renal biopsy biopsies. The results showed that about 34.5% (10/29) of patients had crescent formation at the first renal biopsy. However, some scholars reported that only 11% of patients in Europe (Coppo et al., 2014) had crescent formation, and 48% in China (Zeng et al., 2012). In Japan, up to 63% have crescent formation (Katafuchi et al., 2011). We speculated that this might be due to the fact that the incidence of IgAN in Asian countries is much higher than that in European and American countries. The proportion of results in our study was lower than that reported in the literature, possibly because our study only included patients with repeated renal biopsy. It is known that mesangial cell proliferation, segmental glomerulosclerosis, tubular atrophy and interstitial fibrosis are pathological indicators of poor prognosis (Working Group of the International Ig et al., 2009a; Working Group of the International Ig et al., 2009b). In our data, segmental glomerulosclerosis, globular glomerulosclerosis, and renal interstitial fibrosis could be found after treatment. The index of acute and chronic lesions always presented, indicating that IgAN patients with isolated hematuria were not expected as a good prognosis traditionally. It has been reported that there is a significant relationship between hematuria and the increased cells in capillaries and segmental glomerulosclerosis (Sevillano et al., 2017). Therefore, hematuria can be regarded as a biomarker of activity and severity of renal tissue injury of IgAN.

In recent years, many clinicians have paid attention to IgAN patients with hematuria. In a retrospective cohort study of 1,333 patients with IgAN, professor Zhang found that remission of hematuria within 6 months after diagnosis was associated with a significant reduction in the incidence of renal disease progression events (Yu et al., 2020). There was a significant interaction between remission of proteinuria and remission of hematuria in the 6 months prior to treatment. A meta-analysis of 5,660 IgAN patients from 13 literatures (He et al., 2021) concluded that initial microscopic hematuria and persistent hematuria may be associated with renal progression and ESRD. A Kuwaiti study of 69 IgAN patients (Ghani et al., 2011) showed that renal function deterioration was more significant during follow-up (mean follow-up 3.5 years) in the presence of gross hematuria at biopsy. Our center has been paying close attention to IgAN patients with hematuria. In this study, the longest interval of repeated renal biopsy puncture was nearly 10 years, and the average interval was about 3 years. Our clinical data showed no significant differences in blood pressure, renal function, uric acid, albumin, cholesterol, hemoglobin, and urinary protein between the two renal biopsies, except for a significant decrease in urinary red blood cells and triglycerides. We speculated that this might be due to the small number of cases enrolled, short observation period, limited entry conditions, and active treatment.

So far, the mechanism of hematuria has not been elucidated. It has been reported that the mechanism may be due to IgA immune complex deposition, mesangial activation and immune system disorders through mucosa-renal axis (Suzuki et al., 2011) and CX3CL1/CX3CR1 axis (Suzuki et al., 2011; Cox et al., 2012) leading to infiltration of inflammatory cells and release of mediators, which ultimately leads to chronic inflammation, glomerular capillary wall damage and red blood cell extravasation, thus resulting in hematuria (Gutierrez et al., 2012a). Therefore, hematuria is a reflection of inflammation (Coppo and Fervenza, 2017). In our data, we found the inflammatory cells infiltrating the tubulointerstitium. Paticularly, CD4^+^ cell infiltration decreased significantly after treatment, indicating that active treatment could reduce the infiltration of CD4^+^ inflammatory cells. Interstingly, we also found podocytes were flat even apoptotic, foot process fusion before treatment. After treatment, the foot cell morphology was improved. The mitochondria in podocytes were indistinct, cristae were broken, and local vacuolization in the first renal biopsy specimen. The shape of mitochondria was improved and the number of mitochondria in podocytes was significantly incresed in the second biopsy. Thus, we speculated that active anti-inflammatory therapy for IgAN patients with hematuria may improve prognosis and podocytes were most likely the therapeutic target. Futher, we found that the oxidative stress products of podocytes decreased significantly after treatment (Figure 7). Therefore, we speculated that the oxidative stress of podocyte was involved in the progress of hematuria in IgAN.

Then how to treat IgAN patients with isolated hematuria? It is not clear yet. NEFIGAN clinical trial showed that budesonide treatment reduced hematuria in patients with IgAN (Fellstrom et al., 2017). Prednisone therapy reduced hematuria in 58% patients (Lv et al., 2017). In a retrospective cohort study, it was demonstrated that immunosuppressants could alleviate hematuria (Yu et al., 2020). Other researchers reported that in patients receiving immunosuppressive therapy, hematuria decreased significantly from 36 (6–100) RBC/HPF to 3 (0–12) RBC/HPF after treatment (*p* = 0.001) (Sevillano et al., 2017). In addition to immunosuppressive agents, Chinese researchers attempted to use renin-angiotensin system blockade (RASB) to treat IgAN patients with isolated hemuria. The follow-up period of 24.0 (12.8–36.3) months showed that RASB could effectively relieve hemuria. Moreover, it can reduce the occurrence of subsequent proteinuria, and not only does not affect the blood pressure of non-hypertensive patients, nor does it affect the GFR reduction rate (Chen et al., 2020). In our study, individualized treatment plans were carried out on patients based on their renal pathology and clinical indicators. In order to avoid adverse drug reactions, low-dose, multi-target regimens were generally selected to mimic the treatment strategy for lupus nephritis (as shown in Table 2). Combined with the immunofluorescence results of IgA and C3 from two renal biopsies, IgA deposition did not decrease significantly, while C3 showed a downward trend, indicating that immune activity continued and immunosuppressants could inhibit the complement activation pathway to some certain extent. The pathological scores of the two renal biopsies showed that the acute lesions after treatment were significantly reduced compared with those before treatment, especially the reduction of cellular and fibrous crescent bodies, indicating that immunosuppressants can effectively inhibit acute inflammation. Unfortunately, in this study the chronic lesions were aggravated after treatment, especially segmental and globular glomerulosclerosis, indicating that immunosuppressive agents could not prevent the patient’s chronic renal progression. From the perspective of clinical indicators, renal function, urinary protein and metabolic indicators of patients were relatively stable during the two renal biopsies, thus indicating that immunosuppressive therapy can stabilize renal function to some extent. This treatment regimen also resulted in significant reduction of hematuria. It should be noted that reduction in hematuria is not necessarily the same as improvement in pathology. It is easy to mask the true condition.

In summary, hematuria exacerbates the progression of kidney disease. In addition to IgAN, hematuria has been confirmed to play an important role in diabetic nephropathy (Okada et al., 2020), lupus nephritis (Ding et al., 2015) and ANCA-associated vasculitis (Rhee et al., 2018). Hematuria is a classic manifestation of IgAN, which should be paid more attention to. Based on the results of our study, we suggest that these patients undergo renal biopsy puncture to clarify the renal pathology, and make individualized treatment plans based on the pathological and clinical conditions. Podocytes are most likely the therapeutic target and oxidative stress pathway is involved. In particular, traditional Chinese medicine (TCM) has summarized many valuable experiences in treating IgAN patients with hematuria according to the principle of syndrome differentiation, which has been confirmed in clinical studies. However, due to the complexity of clinical and pathological manifestations of IgAN and the difficulty in quantifying TCM symptoms and evaluation indexes, it is difficult to evaluate TCM efficacy.

There are several limitations in this study. First, this is a single-center study with small sample size, therefore, the conclusions drawn in this study should be further validated through large-scale studies. Second, this is a retrospective study, and randomized prospective controlled trials are still needed to validate our findings in the further studies. Third, the follow-up period of this study was relatively short, thus long-term follow up of large-scale patients in clinical trials are still needed. Four, the statistical results may be biased when patients choose different treatment strategies. On the other hand, we did not correlate the renal outcomes in the cohort. Last, in this study, due to the limited scope of transmission electron microscope observation, we did not make statistics on mitochondria-related data (mitochondrial dynamics related genes, proteins, etc.), instead only described the morphological changes of mitochondria observed by transmission electron microscope (as shown in Figures 6D, E). We will conduct in-depth research on focused mitochondria in the future.

## Conclusion

More and more studies have confirmed that hematuria reflects progressing of IgAN. Based on the results of repeated renal biopsy, we found that there were inflammatory changes in the pathology of IgAN patients with isolated hematuria, which progressed slowly. The oxidative stress of podocytes was likely to participate in this pathophysiological process. This study provides a strong basis for the progress of isolated hematuria IgAN through pathological changes before and after treatment. This study will help clinicians recognize the harm of hematuria to IgAN, change the traditional treatment concept, and help such patients get early treatment.

## Data Availability

The raw data supporting the conclusion of this article will be made available by the authors, without undue reservation.
